# 2-Methyl-3-(2-methyl­phen­yl)-4-oxo-3,4-dihydro­quinazolin-8-yl 4-bromo­benzene-1-sulfonate

**DOI:** 10.1107/S1600536812006198

**Published:** 2012-02-17

**Authors:** Adel S. El-Azab, Alaa A.-M. Abdel-Aziz, Seik Weng Ng, Edward R. T. Tiekink

**Affiliations:** a Department of Pharmaceutical Chemistry, College of Pharmacy, King Saud University, Riyadh 11451, Saudi Arabia; b Department of Organic Chemistry, Faculty of Pharmacy, Al-Azhar University, Cairo 11884, Egypt; c Department of Medicinal Chemistry, Faculty of Pharmacy, University of Mansoura, Mansoura 35516, Egypt; d Department of Chemistry, University of Malaya, 50603 Kuala Lumpur, Malaysia; e Chemistry Department, Faculty of Science, King Abdulaziz University, PO Box 80203 Jeddah, Saudi Arabia

## Abstract

The title mol­ecule, C_22_H_17_BrN_2_O_4_S, has a twisted U shape, the dihedral angle between the quinazolin-4-one and bromo­benzene ring systems being 46.25 (8)°. In order to avoid steric clashes with adjacent substituents on the quinazolin-4-one ring, the N-bound tolyl group occupies an orthogonal position [dihedral angle = 89.59 (8)°]. In the crystal, mol­ecules are connected into a three-dimensional architecture by C—H⋯O inter­actions, with the ketone O atom accepting two such bonds and a sulfonate O atom one.

## Related literature

For the pharmacological activity of substituted quinazoline-4(3*H*)-ones, see: El-Azab & El-Tahir (2012 ▶); El-Azab *et al.* (2011 ▶); Al-Omary *et al.* (2010 ▶); Al-Obaid *et al.* (2009 ▶); Aziza *et al.* (1996 ▶). For the synthesis and evaluation of the anti-convulsant activity of the title compound, see: El-Azab *et al.* (2010 ▶).
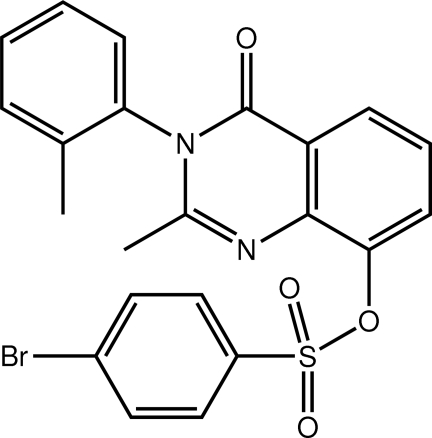


## Experimental

### 

#### Crystal data

C_22_H_17_BrN_2_O_4_S
*M* *_r_* = 485.35
Monoclinic, 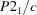

*a* = 11.0587 (3) Å
*b* = 14.4794 (3) Å
*c* = 13.1357 (3) Å
β = 102.804 (2)°
*V* = 2051.03 (8) Å^3^
*Z* = 4
Cu *K*α radiation
μ = 3.96 mm^−1^
*T* = 100 K
0.30 × 0.25 × 0.20 mm

#### Data collection

Agilent SuperNova Dual diffractometer with an Atlas detector
Absorption correction: multi-scan (*CrysAlis PRO*; Agilent, 2011 ▶) *T*_min_ = 0.438, *T*_max_ = 1.000
8236 measured reflections
4208 independent reflections
3952 reflections with *I* > 2σ(*I*)
*R*_int_ = 0.021

#### Refinement

*R*[*F* ^2^ > 2σ(*F* ^2^)] = 0.035
*wR*(*F* ^2^) = 0.098
*S* = 1.07
4208 reflections
273 parameters
H-atom parameters constrained
Δρ_max_ = 0.40 e Å^−3^
Δρ_min_ = −0.95 e Å^−3^

### 

Data collection: *CrysAlis PRO* (Agilent, 2011 ▶); cell refinement: *CrysAlis PRO*; data reduction: *CrysAlis PRO*; program(s) used to solve structure: *SHELXS97* (Sheldrick, 2008 ▶); program(s) used to refine structure: *SHELXL97* (Sheldrick, 2008 ▶); molecular graphics: *ORTEP-3* (Farrugia, 1997 ▶) and *DIAMOND* (Brandenburg, 2006 ▶); software used to prepare material for publication: *publCIF* (Westrip, 2010 ▶).

## Supplementary Material

Crystal structure: contains datablock(s) global, I. DOI: 10.1107/S1600536812006198/hb6634sup1.cif

Structure factors: contains datablock(s) I. DOI: 10.1107/S1600536812006198/hb6634Isup2.hkl

Supplementary material file. DOI: 10.1107/S1600536812006198/hb6634Isup3.cml

Additional supplementary materials:  crystallographic information; 3D view; checkCIF report

## Figures and Tables

**Table 1:** Hydrogen-bond geometry (Å, °)

*D*—H⋯*A*	*D*—H	H⋯*A*	*D*⋯*A*	*D*—H⋯*A*
C3—H3⋯O4^i^	0.95	2.31	3.236 (3)	164
C8—H8⋯O3^ii^	0.95	2.49	3.375 (3)	155
C9—H9⋯O4^iii^	0.95	2.43	3.328 (3)	158

Symmetry codes: (i) 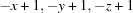; (ii) 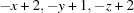; (iii) 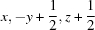.

**Fig. 1.**
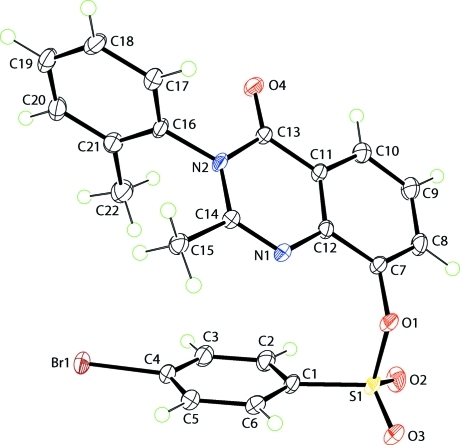
The molecular structure of (I) showing displacement ellipsoids at the 50% probability level.

**Fig. 2.**
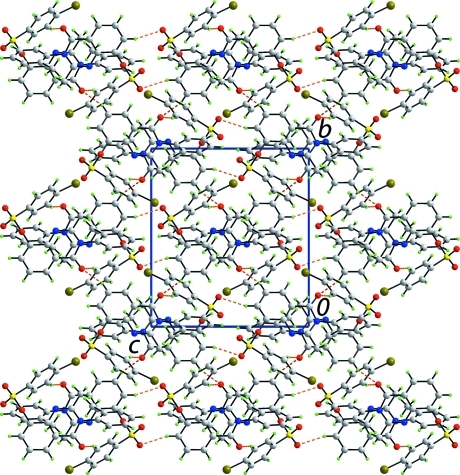
A view in projection down the a axis of the unit-cell contents for (I). The C—H···O interactions are shown as orange dashed lines.

## supplementary crystallographic information

### Comment

The biological activity of substituted quinazoline-4(3*H*)-ones is well documented (El-Azab & El-Tahir, 2012; El-Azab *et al.*, 2011; El-Azab *et al.*, 2010; Al-Omary *et al.*, 2010; Al-Obaid *et al.*, 2009; Aziza *et al.*, 1996). In this connection, the title compound, 3,4-dihydro-2-methyl-3-(2-methylphenyl)-4-oxoquinazolin-8-yl 4-bromobenzenesulfonate (I), a methaqualone analogue, was recently synthesized and evaluated for its anti-convulsant activity (El-Azab *et al.*, 2010). The crystal structure determination of (I) is reported herein.

Overall, the shape of (I), Fig. 1, is of a twisted U as the bromobenzene ring is folded over towards the quinazolin-4-one group. The dihedral angle between the bromobenzene and quinazolin-4-one [r.m.s. deviation = 0.040 Å for the ten atoms] groups is 46.25 (8)°. The dihedral angle between the quinazolin-4-one and *N*-bound tolyl group is 89.59 (8)° indicating an orthogonal arrangement, an orientation which precludes steric clashes with the substituents on the quinazolin-4-one group.

In the crystal packing, C—H···O interactions involving bifurcated ketone-O and one of the sulfonate-O atoms are formed, Table 1. These lead to a three-dimensional architecture, Fig. 2.

### Experimental

A mixture of 8-hydroxymethaqualone (532 mg, 0.0002 *M*) and 4-bromobenzenesulfonyl chloride (534 mg, 0.0021 mmol) in 15 ml pyridine was stirred at room temperature for 11 h. The solvent was removed under reduced pressure, and the residue was triturated with water and filtered. The solid obtained was dried and recrystallized from EtOH. *M*.pt. 451–453 K. Yield: 93%. ^1^H NMR (500 MHz, CDCl_3_): δ = 8.21 (d, 1H, J = 8.0 Hz), 7.80–7.75 (m, 3H), 7.62 (d, 2H, J = 9.0 Hz), 7.48–7.36 (m, 4H), 7.10 (d, 1H, J = 6.5 Hz), 2.05 (s, 3H), 1.95 (s, 3H) p.p.m.. ^13^C NMR (CDCl_3_): δ = 17.2, 23.7, 122.5, 126.4, 127.7, 129.1, 129.3, 129.8, 130.6, 131.6, 132.0, 135.0, 135.1, 136.3, 140.9, 143.6, 155.0, 160.5 p.p.m.. MS (70 eV): m/*z* = 484, 486 (*M*+2).

### Refinement

Carbon-bound H-atoms were placed in calculated positions [C—H = 0.95 to 0.98 Å, *U*_iso_(H) = 1.2–1.5*U*_eq_(C)] and were included in the refinement in the riding model approximation.

### Crystal data

**Table:**

C_22_H_17_BrN_2_O_4_S	*F*(000) = 984
*M**_r_* = 485.35	*D*_x_ = 1.572 Mg m^−^^3^
Monoclinic, *P*2_1_/*c*	Cu *K*α radiation, λ = 1.5418 Å
Hall symbol: -P 2ybc	Cell parameters from 4968 reflections
*a* = 11.0587 (3) Å	θ = 3.1–76.3°
*b* = 14.4794 (3) Å	µ = 3.96 mm^−^^1^
*c* = 13.1357 (3) Å	*T* = 100 K
β = 102.804 (2)°	Block, colourless
*V* = 2051.03 (8) Å^3^	0.30 × 0.25 × 0.20 mm
*Z* = 4	

### Data collection

**Table:**

Agilent SuperNova Dual diffractometer with an Atlas detector	4208 independent reflections
Radiation source: SuperNova (Cu) X-ray Source	3952 reflections with *I* > 2σ(*I*)
Mirror monochromator	*R*_int_ = 0.021
Detector resolution: 10.4041 pixels mm^-1^	θ_max_ = 76.5°, θ_min_ = 4.1°
ω scan	*h* = −13→13
Absorption correction: multi-scan (*CrysAlis PRO*; Agilent, 2011)	*k* = −13→18
*T*_min_ = 0.438, *T*_max_ = 1.000	*l* = −15→16
8236 measured reflections	

### Refinement

**Table:**

Refinement on *F*^2^	Primary atom site location: structure-invariant direct methods
Least-squares matrix: full	Secondary atom site location: difference Fourier map
*R*[*F*^2^ > 2σ(*F*^2^)] = 0.035	Hydrogen site location: inferred from neighbouring sites
*wR*(*F*^2^) = 0.098	H-atom parameters constrained
*S* = 1.07	*w* = 1/[σ^2^(*F*_o_^2^) + (0.0605*P*)^2^ + 1.0031*P*] where *P* = (*F*_o_^2^ + 2*F*_c_^2^)/3
4208 reflections	(Δ/σ)_max_ = 0.001
273 parameters	Δρ_max_ = 0.40 e Å^−^^3^
0 restraints	Δρ_min_ = −0.95 e Å^−^^3^

### Special details

**Table:**

Geometry. All e.s.d.'s (except the e.s.d. in the dihedral angle between two l.s. planes) are estimated using the full covariance matrix. The cell e.s.d.'s are taken into account individually in the estimation of e.s.d.'s in distances, angles and torsion angles; correlations between e.s.d.'s in cell parameters are only used when they are defined by crystal symmetry. An approximate (isotropic) treatment of cell e.s.d.'s is used for estimating e.s.d.'s involving l.s. planes.
Refinement. Refinement of *F*^2^ against ALL reflections. The weighted *R*-factor *wR* and goodness of fit *S* are based on *F*^2^, conventional *R*-factors *R* are based on *F*, with *F* set to zero for negative *F*^2^. The threshold expression of *F*^2^ > σ(*F*^2^) is used only for calculating *R*-factors(gt) *etc*. and is not relevant to the choice of reflections for refinement. *R*-factors based on *F*^2^ are statistically about twice as large as those based on *F*, and *R*- factors based on ALL data will be even larger.

### Fractional atomic coordinates and isotropic or equivalent isotropic displacement parameters (Å^2^)

**Table:**

	*x*	*y*	*z*	*U*_iso_*/*U*_eq_	
Br1	0.55786 (2)	0.803520 (16)	0.479620 (16)	0.02586 (10)	
O1	0.95920 (14)	0.53185 (10)	0.82122 (10)	0.0193 (3)	
O2	0.81926 (17)	0.56446 (12)	0.93880 (12)	0.0291 (4)	
O3	0.99573 (17)	0.66569 (12)	0.92714 (12)	0.0291 (4)	
S1	0.89405 (5)	0.60873 (4)	0.87820 (3)	0.02064 (13)	
N1	0.88588 (16)	0.53757 (12)	0.60644 (12)	0.0162 (3)	
N2	0.77713 (16)	0.46955 (12)	0.44884 (12)	0.0158 (3)	
O4	0.69320 (15)	0.32500 (10)	0.44155 (11)	0.0204 (3)	
C1	0.7994 (2)	0.66819 (15)	0.77401 (15)	0.0188 (4)	
C2	0.6754 (2)	0.64461 (15)	0.74360 (16)	0.0218 (4)	
H2	0.6407	0.6000	0.7820	0.026*	
C3	0.6019 (2)	0.68678 (16)	0.65633 (17)	0.0227 (4)	
H3	0.5164	0.6719	0.6342	0.027*	
C4	0.6564 (2)	0.75095 (15)	0.60266 (15)	0.0205 (4)	
C5	0.7798 (2)	0.77732 (15)	0.63407 (16)	0.0209 (4)	
H5	0.8135	0.8234	0.5969	0.025*	
C6	0.8529 (2)	0.73486 (15)	0.72109 (15)	0.0202 (4)	
H6	0.9378	0.7509	0.7442	0.024*	
C7	0.88853 (19)	0.45840 (14)	0.76930 (15)	0.0174 (4)	
C8	0.8594 (2)	0.38427 (15)	0.82488 (16)	0.0220 (4)	
H8	0.8827	0.3844	0.8990	0.026*	
C9	0.7954 (2)	0.30868 (15)	0.77233 (18)	0.0240 (5)	
H9	0.7749	0.2577	0.8108	0.029*	
C10	0.7620 (2)	0.30825 (14)	0.66447 (17)	0.0211 (4)	
H10	0.7197	0.2567	0.6284	0.025*	
C11	0.79106 (19)	0.38491 (14)	0.60856 (15)	0.0162 (4)	
C12	0.85457 (19)	0.46112 (14)	0.65901 (15)	0.0154 (4)	
C13	0.74931 (19)	0.38693 (14)	0.49465 (15)	0.0159 (4)	
C14	0.84603 (19)	0.53990 (14)	0.50603 (15)	0.0163 (4)	
C15	0.8748 (2)	0.62223 (14)	0.44701 (16)	0.0203 (4)	
H15A	0.9282	0.6647	0.4951	0.030*	
H15B	0.7975	0.6537	0.4143	0.030*	
H15C	0.9175	0.6023	0.3929	0.030*	
C16	0.7300 (2)	0.47748 (14)	0.33657 (15)	0.0171 (4)	
C17	0.8015 (2)	0.44417 (15)	0.27003 (16)	0.0194 (4)	
H17	0.8806	0.4178	0.2976	0.023*	
C18	0.7562 (2)	0.44979 (15)	0.16290 (16)	0.0227 (4)	
H18	0.8041	0.4272	0.1165	0.027*	
C19	0.6406 (2)	0.48863 (16)	0.12407 (16)	0.0234 (4)	
H19	0.6092	0.4925	0.0508	0.028*	
C20	0.5708 (2)	0.52162 (15)	0.19111 (16)	0.0226 (4)	
H20	0.4922	0.5486	0.1630	0.027*	
C21	0.6131 (2)	0.51634 (14)	0.29958 (16)	0.0202 (4)	
C22	0.5361 (2)	0.55171 (18)	0.37228 (18)	0.0282 (5)	
H22A	0.5849	0.5957	0.4215	0.042*	
H22B	0.5110	0.4999	0.4109	0.042*	
H22C	0.4621	0.5826	0.3318	0.042*	

### Atomic displacement parameters (Å^2^)

**Table:**

	*U*^11^	*U*^22^	*U*^33^	*U*^12^	*U*^13^	*U*^23^
Br1	0.03211 (16)	0.02299 (14)	0.01724 (14)	0.00025 (8)	−0.00581 (10)	0.00386 (8)
O1	0.0227 (8)	0.0226 (7)	0.0111 (6)	0.0019 (6)	0.0006 (5)	−0.0025 (5)
O2	0.0416 (10)	0.0338 (9)	0.0140 (7)	0.0121 (7)	0.0107 (7)	0.0059 (6)
O3	0.0360 (10)	0.0279 (8)	0.0169 (7)	0.0043 (7)	−0.0078 (6)	−0.0064 (6)
S1	0.0284 (3)	0.0235 (3)	0.0082 (2)	0.0058 (2)	0.00012 (19)	−0.00128 (17)
N1	0.0200 (8)	0.0172 (8)	0.0105 (7)	0.0003 (6)	0.0018 (6)	−0.0006 (6)
N2	0.0194 (8)	0.0189 (8)	0.0083 (7)	−0.0006 (7)	0.0011 (6)	0.0006 (6)
O4	0.0266 (8)	0.0194 (7)	0.0141 (6)	−0.0042 (6)	0.0021 (6)	−0.0016 (5)
C1	0.0231 (11)	0.0218 (10)	0.0101 (8)	0.0044 (8)	0.0006 (7)	−0.0007 (7)
C2	0.0259 (11)	0.0241 (10)	0.0153 (9)	−0.0011 (8)	0.0039 (8)	0.0015 (8)
C3	0.0222 (11)	0.0259 (11)	0.0182 (10)	0.0001 (8)	0.0005 (8)	0.0000 (8)
C4	0.0265 (11)	0.0200 (9)	0.0128 (8)	0.0048 (8)	−0.0004 (8)	−0.0003 (7)
C5	0.0270 (11)	0.0203 (9)	0.0144 (9)	0.0013 (8)	0.0027 (8)	0.0002 (8)
C6	0.0232 (11)	0.0211 (10)	0.0149 (9)	0.0009 (8)	0.0014 (8)	−0.0031 (8)
C7	0.0199 (10)	0.0200 (9)	0.0114 (9)	0.0032 (8)	0.0013 (7)	−0.0017 (7)
C8	0.0271 (11)	0.0261 (11)	0.0124 (9)	0.0053 (9)	0.0035 (8)	0.0047 (8)
C9	0.0314 (12)	0.0227 (11)	0.0175 (10)	−0.0004 (9)	0.0047 (9)	0.0066 (8)
C10	0.0254 (11)	0.0197 (10)	0.0175 (10)	−0.0005 (8)	0.0035 (8)	0.0019 (8)
C11	0.0188 (10)	0.0182 (9)	0.0109 (8)	0.0027 (7)	0.0018 (7)	0.0007 (7)
C12	0.0174 (9)	0.0174 (9)	0.0113 (8)	0.0031 (7)	0.0027 (7)	0.0019 (7)
C13	0.0185 (10)	0.0174 (9)	0.0113 (9)	0.0022 (7)	0.0026 (7)	−0.0001 (7)
C14	0.0188 (9)	0.0169 (9)	0.0127 (8)	0.0010 (7)	0.0023 (7)	−0.0002 (7)
C15	0.0267 (11)	0.0187 (10)	0.0149 (9)	−0.0039 (8)	0.0032 (8)	0.0015 (7)
C16	0.0222 (10)	0.0181 (9)	0.0093 (8)	−0.0027 (8)	−0.0005 (7)	0.0015 (7)
C17	0.0204 (10)	0.0225 (10)	0.0142 (9)	−0.0004 (8)	0.0015 (8)	0.0011 (7)
C18	0.0287 (11)	0.0265 (11)	0.0128 (9)	−0.0025 (9)	0.0047 (8)	−0.0034 (8)
C19	0.0275 (12)	0.0265 (11)	0.0131 (9)	−0.0071 (9)	−0.0025 (8)	0.0021 (8)
C20	0.0241 (11)	0.0233 (10)	0.0178 (10)	−0.0019 (8)	−0.0010 (8)	0.0043 (8)
C21	0.0223 (10)	0.0200 (10)	0.0175 (9)	−0.0009 (8)	0.0028 (8)	0.0005 (8)
C22	0.0298 (12)	0.0326 (12)	0.0219 (10)	0.0056 (10)	0.0054 (9)	−0.0007 (9)

### Geometric parameters (Å, º)

**Table:**

Br1—C4	1.897 (2)	C8—H8	0.9500
O1—C7	1.404 (2)	C9—C10	1.383 (3)
O1—S1	1.5991 (15)	C9—H9	0.9500
O2—S1	1.4214 (18)	C10—C11	1.407 (3)
O3—S1	1.4273 (18)	C10—H10	0.9500
S1—C1	1.754 (2)	C11—C12	1.394 (3)
N1—C14	1.295 (2)	C11—C13	1.465 (3)
N1—C12	1.388 (3)	C14—C15	1.494 (3)
N2—C14	1.389 (3)	C15—H15A	0.9800
N2—C13	1.403 (3)	C15—H15B	0.9800
N2—C16	1.456 (2)	C15—H15C	0.9800
O4—C13	1.218 (3)	C16—C21	1.394 (3)
C1—C2	1.384 (3)	C16—C17	1.389 (3)
C1—C6	1.395 (3)	C17—C18	1.387 (3)
C2—C3	1.391 (3)	C17—H17	0.9500
C2—H2	0.9500	C18—C19	1.387 (3)
C3—C4	1.383 (3)	C18—H18	0.9500
C3—H3	0.9500	C19—C20	1.378 (3)
C4—C5	1.389 (3)	C19—H19	0.9500
C5—C6	1.389 (3)	C20—C21	1.400 (3)
C5—H5	0.9500	C20—H20	0.9500
C6—H6	0.9500	C21—C22	1.504 (3)
C7—C8	1.376 (3)	C22—H22A	0.9800
C7—C12	1.414 (3)	C22—H22B	0.9800
C8—C9	1.399 (3)	C22—H22C	0.9800
			
C7—O1—S1	119.65 (13)	C12—C11—C13	118.76 (18)
O2—S1—O3	120.43 (10)	C10—C11—C13	119.48 (19)
O2—S1—O1	109.07 (9)	N1—C12—C11	123.33 (17)
O3—S1—O1	102.79 (9)	N1—C12—C7	119.52 (18)
O2—S1—C1	109.73 (10)	C11—C12—C7	117.15 (18)
O3—S1—C1	110.01 (10)	O4—C13—N2	120.95 (17)
O1—S1—C1	103.26 (9)	O4—C13—C11	125.12 (19)
C14—N1—C12	117.46 (18)	N2—C13—C11	113.92 (17)
C14—N2—C13	122.64 (16)	N1—C14—N2	123.62 (18)
C14—N2—C16	121.36 (16)	N1—C14—C15	119.06 (18)
C13—N2—C16	115.99 (16)	N2—C14—C15	117.32 (17)
C2—C1—C6	121.96 (19)	C14—C15—H15A	109.5
C2—C1—S1	119.09 (17)	C14—C15—H15B	109.5
C6—C1—S1	118.87 (16)	H15A—C15—H15B	109.5
C1—C2—C3	119.4 (2)	C14—C15—H15C	109.5
C1—C2—H2	120.3	H15A—C15—H15C	109.5
C3—C2—H2	120.3	H15B—C15—H15C	109.5
C4—C3—C2	118.3 (2)	C21—C16—C17	122.27 (18)
C4—C3—H3	120.9	C21—C16—N2	118.76 (18)
C2—C3—H3	120.9	C17—C16—N2	118.95 (18)
C3—C4—C5	122.96 (19)	C18—C17—C16	119.4 (2)
C3—C4—Br1	118.08 (17)	C18—C17—H17	120.3
C5—C4—Br1	118.95 (16)	C16—C17—H17	120.3
C4—C5—C6	118.5 (2)	C17—C18—C19	119.5 (2)
C4—C5—H5	120.8	C17—C18—H18	120.2
C6—C5—H5	120.8	C19—C18—H18	120.2
C5—C6—C1	118.9 (2)	C20—C19—C18	120.44 (19)
C5—C6—H6	120.6	C20—C19—H19	119.8
C1—C6—H6	120.6	C18—C19—H19	119.8
C8—C7—O1	120.25 (17)	C19—C20—C21	121.6 (2)
C8—C7—C12	121.67 (19)	C19—C20—H20	119.2
O1—C7—C12	118.00 (18)	C21—C20—H20	119.2
C7—C8—C9	120.04 (19)	C16—C21—C20	116.9 (2)
C7—C8—H8	120.0	C16—C21—C22	121.87 (19)
C9—C8—H8	120.0	C20—C21—C22	121.3 (2)
C10—C9—C8	120.0 (2)	C21—C22—H22A	109.5
C10—C9—H9	120.0	C21—C22—H22B	109.5
C8—C9—H9	120.0	H22A—C22—H22B	109.5
C9—C10—C11	119.4 (2)	C21—C22—H22C	109.5
C9—C10—H10	120.3	H22A—C22—H22C	109.5
C11—C10—H10	120.3	H22B—C22—H22C	109.5
C12—C11—C10	121.71 (18)		
			
C7—O1—S1—O2	46.87 (16)	C8—C7—C12—N1	179.87 (19)
C7—O1—S1—O3	175.77 (14)	O1—C7—C12—N1	−3.4 (3)
C7—O1—S1—C1	−69.77 (16)	C8—C7—C12—C11	−0.6 (3)
O2—S1—C1—C2	−19.3 (2)	O1—C7—C12—C11	176.13 (17)
O3—S1—C1—C2	−153.96 (17)	C14—N2—C13—O4	−176.82 (19)
O1—S1—C1—C2	96.90 (18)	C16—N2—C13—O4	2.8 (3)
O2—S1—C1—C6	163.76 (16)	C14—N2—C13—C11	4.5 (3)
O3—S1—C1—C6	29.1 (2)	C16—N2—C13—C11	−175.88 (17)
O1—S1—C1—C6	−80.07 (18)	C12—C11—C13—O4	180.0 (2)
C6—C1—C2—C3	1.5 (3)	C10—C11—C13—O4	−2.6 (3)
S1—C1—C2—C3	−175.36 (17)	C12—C11—C13—N2	−1.4 (3)
C1—C2—C3—C4	0.3 (3)	C10—C11—C13—N2	176.04 (18)
C2—C3—C4—C5	−2.3 (3)	C12—N1—C14—N2	−1.7 (3)
C2—C3—C4—Br1	176.82 (16)	C12—N1—C14—C15	178.31 (18)
C3—C4—C5—C6	2.4 (3)	C13—N2—C14—N1	−3.1 (3)
Br1—C4—C5—C6	−176.64 (15)	C16—N2—C14—N1	177.25 (19)
C4—C5—C6—C1	−0.6 (3)	C13—N2—C14—C15	176.87 (18)
C2—C1—C6—C5	−1.3 (3)	C16—N2—C14—C15	−2.7 (3)
S1—C1—C6—C5	175.54 (16)	C14—N2—C16—C21	−89.8 (2)
S1—O1—C7—C8	−79.5 (2)	C13—N2—C16—C21	90.6 (2)
S1—O1—C7—C12	103.79 (18)	C14—N2—C16—C17	91.7 (2)
O1—C7—C8—C9	−176.1 (2)	C13—N2—C16—C17	−87.9 (2)
C12—C7—C8—C9	0.5 (3)	C21—C16—C17—C18	0.3 (3)
C7—C8—C9—C10	0.3 (3)	N2—C16—C17—C18	178.74 (19)
C8—C9—C10—C11	−1.0 (3)	C16—C17—C18—C19	0.1 (3)
C9—C10—C11—C12	0.9 (3)	C17—C18—C19—C20	0.1 (3)
C9—C10—C11—C13	−176.4 (2)	C18—C19—C20—C21	−0.7 (3)
C14—N1—C12—C11	4.9 (3)	C17—C16—C21—C20	−0.8 (3)
C14—N1—C12—C7	−175.62 (19)	N2—C16—C21—C20	−179.26 (18)
C10—C11—C12—N1	179.4 (2)	C17—C16—C21—C22	179.6 (2)
C13—C11—C12—N1	−3.2 (3)	N2—C16—C21—C22	1.2 (3)
C10—C11—C12—C7	−0.1 (3)	C19—C20—C21—C16	1.0 (3)
C13—C11—C12—C7	177.24 (18)	C19—C20—C21—C22	−179.4 (2)

### Hydrogen-bond geometry (Å, º)

**Table:**

*D*—H···*A*	*D*—H	H···*A*	*D*···*A*	*D*—H···*A*
C3—H3···O4^i^	0.95	2.31	3.236 (3)	164
C8—H8···O3^ii^	0.95	2.49	3.375 (3)	155
C9—H9···O4^iii^	0.95	2.43	3.328 (3)	158

Symmetry codes: (i) −*x*+1, −*y*+1, −*z*+1; (ii) −*x*+2, −*y*+1, −*z*+2; (iii) *x*, −*y*+1/2, *z*+1/2.

## References

[bb1] Agilent (2011). *CrysAlis PRO* Agilent Technologies, Yarnton, Oxfordshire, England.

[bb2] Al-Obaid, A. M., Abdel-Hamide, S. G., El-Kashef, H. A., Abdel-Aziz, A. A.-M., El-Azab, A. S., Al-Khamees, H. A. & El-Subbagh, H. I. (2009). *Eur. J. Med. Chem.* **44**, 2379–2391. 10.1016/j.ejmech.2008.09.015 18950904

[bb3] Al-Omary, F. A., Abou-Zeid, L. A., Nagi, M. N., Habib, S. E., Abdel-Aziz, A. A.-M., Hamide, S. G., Al-Omar, M. A., Al-Obaid, A. M. & El-Subbagh, H. I. (2010). *Bioorg. Med. Chem.* **18**, 2849–2863. 10.1016/j.bmc.2010.03.019 20350811

[bb4] Aziza, M. A., Nassar, M. W. I., Abdel Hamid, S. G., El-Hakim, A. E. & El-Azab, A. S. (1996). *Indian J. Heterocycl. Chem* **6**, 25–30.

[bb5] Brandenburg, K. (2006). *DIAMOND* Crystal Impact GbR, Bonn, Germany.

[bb6] El-Azab, A. S., Al-Omar, M. A., Abdel-Aziz, A. A.-M., Abdel-Aziz, N. I., El-Sayed, M. A.-A., Aleisa, A. M., Sayed-Ahmed, M. M. & Abdel-Hamide, S. G. (2010). *Eur. J. Med. Chem.* **45**, 4188–4198. 10.1016/j.ejmech.2010.06.013 20599299

[bb7] El-Azab, A. S. & El-Tahir, K. H. (2012). *Bioorg. Med. Chem. Lett.* **22**, 327–333.

[bb8] El-Azab, A. S., El-Tahir, K. H. & Attia, S. M. (2011). *Monatsh. Chem.* **142**, 837–848.

[bb9] Farrugia, L. J. (1997). *J. Appl. Cryst.* **30**, 565.

[bb10] Sheldrick, G. M. (2008). *Acta Cryst.* A**64**, 112–122. 10.1107/S0108767307043930 18156677

[bb11] Westrip, S. P. (2010). *J. Appl. Cryst.* **43**, 920–925.

